# Magnetic resonance cholangiopancreatography enhanced by virtual reality as a novel tool to improve the understanding of biliary anatomy and the teaching of surgical trainees

**DOI:** 10.3389/fsurg.2022.916443

**Published:** 2022-08-12

**Authors:** Sebastian M. Staubli, Peter Maloca, Christoph Kuemmerli, Julia Kunz, Amanda S. Dirnberger, Andreas Allemann, Julian Gehweiler, Savas Soysal, Raoul Droeser, Silvio Däster, Gabriel Hess, Dimitri Raptis, Otto Kollmar, Markus von Flüe, Martin Bolli, Philippe Cattin

**Affiliations:** ^1^ Clarunis, University Center for Gastrointestinal and Liver Diseases, St. Clara Hospital and University Hospital Basel, Basel, Switzerland; ^2^ Clinical Service of HPB Surgery and Liver Transplantation, Royal Free London Hospital, NHS Foundation Trust, London, United Kingdom; ^3^ Department of Ophthalmology, University of Basel, Basel, Switzerland; ^4^ Institute of Molecular and Clinical Ophthalmology Basel (IOB), Basel, Switzerland; ^5^ Moorfields Eye Hospital NHS Foundation Trust, London, United Kingdom; ^6^ Faculty of Medicine, University of Basel, Basel, Switzerland; ^7^ Department of Radiology, University Hospital Basel, Basel, Switzerland; ^8^ Department of Biomedical Engineering, University of Basel, Allschwil, Switzerland

**Keywords:** magnetic resonance cholangiopancreaticography (MRCP), minimally invasive cholec, bile duct anatomy, surgical skills training, immersive virtual reality, 3D printing

## Abstract

**Objective:**

The novel picture archiving and communication system (PACS), compatible with virtual reality (VR) software, displays cross-sectional images in VR. VR magnetic resonance cholangiopancreatography (MRCP) was tested to improve the anatomical understanding and intraoperative performance of minimally invasive cholecystectomy (CHE) in surgical trainees.

**Design:**

We used an immersive VR environment to display volumetric MRCP data (Specto VR^TM^). First, we evaluated the tolerability and comprehensibility of anatomy with a validated simulator sickness questionnaire (SSQ) and examined anatomical landmarks. Second, we compared conventional MRCP and VR MRCP by matching three-dimensional (3D) printed models and identifying and measuring common bile duct stones (CBDS) using VR MRCP. Third, surgical trainees prepared for CHE with either conventional MRCP or VR MRCP, and we measured perioperative parameters and surgical performance (validated GOALS score).

**Setting:**

The study was conducted out at Clarunis, University Center for Gastrointestinal and Liver Disease, Basel, Switzerland.

**Participants:**

For the first and second study step, doctors from all specialties and years of experience could participate. In the third study step, exclusively surgical trainees were included. Of 74 participating clinicians, 34, 27, and 13 contributed data to the first, second, and third study phases, respectively.

**Results:**

All participants determined the relevant biliary structures with VR MRCP. The median SSQ score was 0.75 (IQR: 0, 3.5), indicating good tolerability. Participants selected the corresponding 3D printed model faster and more reliably when previously studying VR MRCP compared to conventional MRCP: We obtained a median of 90 s (IQR: 55, 150) and 72.7% correct answers with VR MRCP versus 150 s (IQR: 100, 208) and 49.6% correct answers with conventional MRCP, respectively (*p* < 0.001). CBDS was correctly identified in 90.5% of VR MRCP cases. The median GOALS score was higher after preparation with VR MRCP than with conventional MRCP for CHE: 16 (IQR: 13, 22) and 11 (IQR: 11, 18), respectively (*p* = 0.27).

**Conclusions:**

VR MRCP allows for a faster, more accurate understanding of displayed anatomy than conventional MRCP and potentially leads to improved surgical performance in CHE in surgical trainees.

## Introduction

Virtual reality (VR) environments allow a near-real-world perception of computer-generated environments and are currently studied as tools to enhance medical imaging for teaching, research, and clinical purposes (1–3).

Medical-grade immersive VR software (Specto VR™) has recently been developed to directly extract datasets from the picture archiving and communication system (PACS) archive and render cross-sectional imaging, such as computed tomography (CT) and magnetic resonance scans (MR), in real time and displayed as interactive three-dimensional (3D) models. A conventional static medical image is transformed from the PACS archive into a 3D virtual space that allows free interaction with the displayed model, including walking into an image, rotating, zooming, and using cutting planes to explore the original dataset in real time (2).

Minimally invasive cholecystectomy (CHE) is performed frequently and is used as a practice operation for trainee surgeons (4). Achieving a 3D understanding of the gallbladder and the biliary anatomy is a prerequisite for performing CHE safely but is often challenging for inexperienced surgeons (5). Common bile duct (CBD) injury (a complication of CHE) can be a devastating consequence of an insufficient understanding of the biliary anatomy. Magnetic resonance cholangiopancreatography (MRCP) depicts biliary anatomy and can be used prior to CHEs to study patients' anatomy (6, 7).

Although the exact number of CHEs needed to fulfil the learning curve is unclear (8), there is a broad consensus that precise knowledge of anatomy and the pitfalls and key surgical phases of CHE improves patient safety (9, 10). Enhancing MRCP with VR is an entirely new 3D approach to improving understanding of the biliary anatomy, potentially allowing for better teaching and preparation before CHEs, with the goal of ultimately improving surgical outcomes and patient safety.

Because this technology is innovative and understudied, we aimed to lay a cornerstone for the future use of VR MRCP as a teaching and surgical preparation tool. We first assessed the tolerability of VR MRCP and the comprehensibility of the displayed biliary anatomy. Subsequently, we directly compared VR MRCP models to conventional MRCP PACS image viewing by comparing both to 3D printed MRCP models. To assess the diagnostic value of VR MRCP imaging according to pathological findings, MRCP images with common bile duct stones (CBDS) were presented to the study participants. Finally, surgical trainees performed CHEs after preparing for surgery using VR or conventional MRCP, allowing us to study the clinical potential of this technology. This is the first study to use VR technology to display MRCP imaging in real time and assess its potential clinical value.

## Materials and methods

This prospective study was carried out at Clarunis, University Center for Gastrointestinal and Liver Disease, Basel, Switzerland. Clarunis consists of the abdominal surgery units of St. Claraspital (SCS) and the University Hospital in Basel (USB), Switzerland. This research was conducted according to the Declaration of Helsinki. The use of patient data was approved by the local Ethics Committee of Northwestern and Central Switzerland (Ethikkommission Nordwest und Zentralschweiz, EKNZ 2021-00457; AO_2021-00053). The study participants gave their written informed consent. Medical data were used if patients had signed our institution's general consent form. If general consent forms were missing, participants signed a written informed consent form issued by the EKNZ before contributing their data.

### Participants

**Figure 1:**
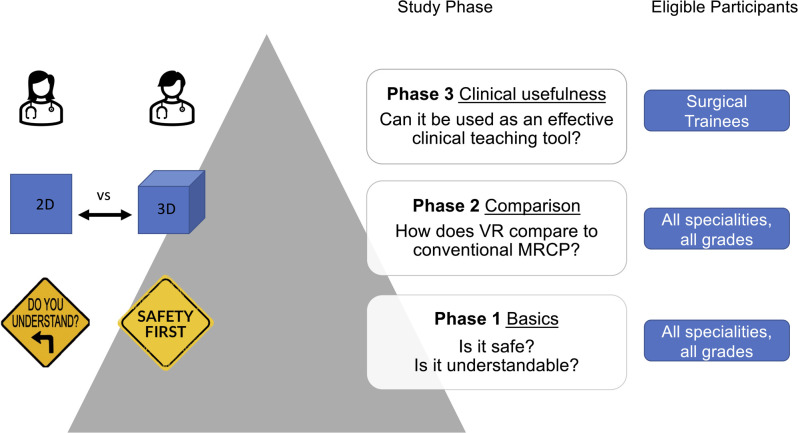
Phasewise study set up. First phase: Establishing the basics of VR MRCP experiences regarding the tolerability and overall safety of the VR experience and understanding of the displayed anatomy. Second phase: Direct comparison of conventional and VR MRCP's ability to demonstrate pathological findings. Third phase: Assessing the potential clinical usefulness of VR MRCP.

Clinicians working as surgeons, gastroenterologists, radiologists, or physicians were eligible for inclusion in the first two study phases. For the third study phase, only surgical trainees who had not completed their surgical training were eligible (Figure 1). Participants received written or personal invitations to participate *via* the clinical departments. Participation was voluntary, and no financial compensation was offered. The participants were verbally informed about the study prior to giving informed consent. Upon inclusion, a personal identification number was assigned to each participant. The number of study participants was determined based on the literature on usability testing (11). To reduce potentially biased outcomes, the participants were not allowed to observe other participants completing the VR experiments.

### Procedures

This study consisted of three separate phases. The first phase assessed user tolerability and safety and the comprehensibility of the VR-displayed anatomy. The second phase directly compared conventional and VR MRCP for facilitating a 3D understanding of anatomy. The third phase assessed the usefulness of this technology as a teaching and preparation tool for CHE (Figure 1).

In the first phase, a pre-test survey included questions regarding demographic data, years of clinical experience, type of training and specialization, and previous VR experience. A head-mounted display (HMD) was fitted to the participants' heads, displaying a virtual room. After acclimatization to the VR surroundings, the participants received instructions about the system. We displayed 3D MRCP models, then asked the users to highlight the relevant anatomy of the biliary system in the displayed MRCP model by pointing the cursor to and naming the structure. The study personnel monitored all actions in the VR scenario on a separate screen. After completing the VR experiments, we assessed tolerability using the validated Simulator Sickness Questionnaire (SSQ), which included questions regarding symptoms of discomfort, dizziness, fatigue, and others (12). A post-test survey was administered immediately after the VR experience, including 17 questions with a 5-point Likert scale (1 = strongly disagree, 2 = disagree, 3 = agree, 4 = strongly agree, 5 = no answer; see Supplementary S1 and S2) inquiring about the handling of the VR system, the understandability of the displayed model and anatomy, the likelihood of using a VR system on a regular basis in a clinical setting, and the expected utility as a tool for planning surgical procedures and improving understanding of anatomy.

**Figure 2:**
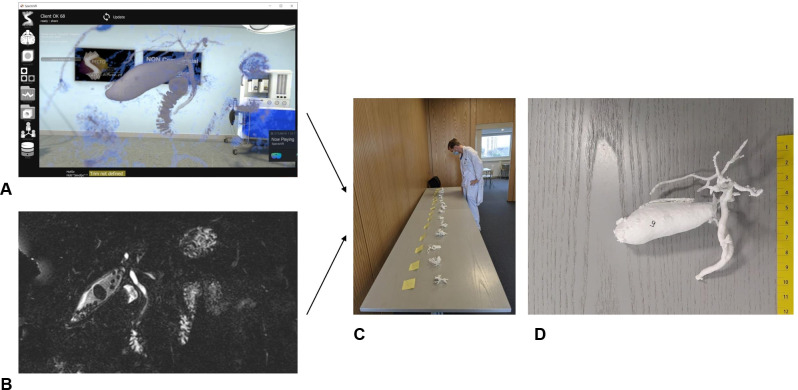
(**A**) A 3D MRCP reconstructed scan viewed by a participant in a virtual room compared to (**B**) a conventional digital imaging and communications in medicine (DICOM)-viewed MRCP scan. Participants were allowed to take as long as necessary to view the scans in the respective modality. Fifteen MRCP scans were 3D printed, and the candidates were asked to choose the correct model (**C,D**).

In the second phase, we compared conventional MRCP and VR MRCP. For this purpose, we presented 15 numbered 3D-printed MRCP models. All 15 scans existed as conventional MRCP and VR MRCP datasets. Participants were randomly assigned to use VR or conventional MRCP imaging, enabling us to distribute the learning effects equally in both modality groups. They were allowed to inspect the MRCP scans with the respective technology with no time limit, and the time taken was measured from the moment they started viewing the image until they stopped. In the conventional MRCP group, the participants were allowed free choice of plane (sagittal, coronal or transverse view). 3D functionality was not included in the conventional imaging display. The participants were asked to choose the corresponding 3D printed model. This procedure was repeated five times for both imaging modalities (VR MRCP and conventional MRCP) with randomly chosen MRCPs in random order. We documented the time needed to select the corresponding model and the correctness of the given answers (Figure 2). VR MRCP scans of CBDS were assessed. Each participant was presented with five VR MRCP scans of CBDS from a total of the 15 previously used scans. The sequence and selection of scans were random. We asked the participants to assess and demonstrate the presence of stones in the VR model and to indicate the location of CBDS in the 3D printed model. After all VR experiments, we asked participants to complete a post-test survey to assess the usefulness of VR MRCP compared to conventional MRCP (Supplementary S2).

In the third study phase, trainee surgeons were randomly assigned to conventional or VR MRCP imaging one day prior to elective CHE. At our institution, MRCP is routinely performed on all patients who undergo elective CHE. We administered a pre-test survey regarding demographic data, years of clinical experience, type of training, and previous surgical experience. Participants were allowed to study the patient's MRCP with no time limit, and the time taken was measured from the moment they started viewing the image until they stopped. We predicted the difficulty of cholecystectomy using a scoring system validated by Gupta et al. (0 = very easy to 15 = very difficult) (13). Participants performed CHEs under the supervision of a board-certified surgeon the following day. The actual difficulty of the procedure was measured with the validated Nassar score (grade I for easiest to grade V for most difficult) (14). We assessed the time to reach the critical view of safety (CVS) and the scoring of the CVS using the validated Sanford-Strasberg CVS score *via* video analysis of the procedure (from 0 [CVS not achieved] to 6 [CVS fully achieved]) (15). Two surgeons assigned the video analysis ratings independently. The trainee surgeons (study participants) and the instructing surgeon, the latter being blinded to study allocation, used the validated GOALS score (5 = least skillful to 25 = most skillful) (16) to assess intraoperative performance (Supplementry S3 and S4).

In the first study phase, blinding was not possible, but in the second and third phases, the participants and the principal investigators were blind to the correctness of the answers. Blinding to the technology used to view MRCP imaging in the second and third study phases was not possible.

### MRCP acquisition

In St. Clara Hospital (SCS), MRCP imaging is routinely acquired for patients who undergo CHEs, following an in-house study that provided evidence of silent CBDS and low postoperative morbidity in patients who underwent MRCP prior to routine CHE (17). At the University Hospital Basel, only patients with suspected CBDS undergo routine MRCP prior to CHEs; therefore, only patients at SCS were included in this study. We anonymized all utilized scans, and for the first study phase, we selected normal MRCP scans. For the second phase, we selected scans displaying CBDS, but excluded scans with pathological findings other than CBDS, insufficient image quality, or anatomical variations. For the third phase, we used scans of patients scheduled for CHEs. We acquired MRCP imaging using Magnetom Avanto Fit (Siemens Healthcare): field strength (1.5 Tesla), isotropic T2-weighted fat-saturated, turbo spin-echo sequence (SPACE), TR (2,500 ms), TE (704 ms), matrix size (640 × 640), voxel size (0.56 × 0.56 × 1 mm^3^), and field of view (360 × 360 mm).

### VR software and equipment

**Figure 3:**
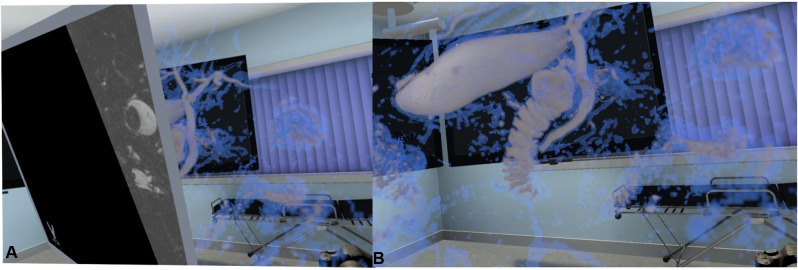
The 3D MRCP model in the VR environment as seen by the viewer. (**A**) The model can be rotated, zoomed, moved freely, and displayed as a VR medical examination room. (**B**) By using the freely adjustable cutting plane, the original scan can be seen through the model.

For the virtual representation of the medical data, we used a VR application, which enabled the importation and display of data in real time and was enhanced with ray casting (Specto VR™, Version 4.0, Specto Medical, Basel, Switzerland). Specto VR™ uses volume rendering at 180 frames/s to visualize medical data in a VR environment. The user can select visualizations of different tissues *via* a freely configurable transfer function. A freely adjustable cutting plane displaying the original dataset (cross-sectional imaging) allows interaction with the original imaging display (Figure 3B). The VR model can be freely rotated in all directions as well as zoomed or miniaturized. The software was run on a laptop computer, ASUS ROG Zephyrus GX501GI-EI005T (15.60″; full HD; Intel Core i7-8750H, 16GB; 512GB hard-drive; and graphics processing unit Nvidia GTX1080 MaxQ), with a VR head-mounted display (HTC Vive, Xindian District, New Taipei City, Taiwan) and HP Mixed Reality (Hewlett-Packard, Palo Alto, California, United States).

### 3D MRCP printing

For 3D printing, anatomical models of the biliary system were segmented with Materialise Mimics and 3-matic (Materialise NV, Leuven, Belgium) by manually selecting signal intensity threshold values, followed by manual refinements. The specimens were saved in “.stl” file format. The models were printed on a MakerBot Replicator+ printer (MakerBot Industries, LLC, Brooklyn, NY, USA) with fused filament fabrication (FFF) technology using a white polylactic acid (PLA) filament. Subsequently, manual post-processing was necessary to remove the supporting structures with fine-cutting pliers.

### Statistical analysis

We performed statistical analysis with R v4.1.2 and assessed the normality of data distribution with the Kolmogorov–Smirnov test. Comparisons were made using (1) the chi-squared test or Fisher exact test for categorical data, (2) the unpaired or paired Student's t-test for continuous normally distributed variables, and (3) the Mann–Whitney U-test for continuous non-normally distributed variables. We applied a Spearman rank coefficient and graphically assessed correlations between years of experience, median time to decision, time taken, correct decision, and correct answers. All values are expressed as medians (interquartile ranges [IQRs]), means ± standard deviations, or counts (percentages), unless otherwise specified. A *P* value <0.05 was considered significant.

## Results

### Subjects

The first phase, to assess the tolerability of the VR experience and understandability of the displayed anatomy, was performed with 34 subjects. Participants included 31 (91%) surgeons and 3 (9%) radiologists. The median age was 37.5 years (IQR: 37, 45), and 14 (42%) were female. The study participants had a median work experience of 11 years (IQR: 11, 17.25), 24 (71%) had completed their training, and 9 (26%) had previous exposure to VR.

The second phase, to compare VR and conventional MRCP for the identification of pathology, involved 27 subjects. Participants included 22 (81%) surgeons, 3 (11%) gastroenterologists, 1 (4%) physician, and 1 (4%) radiologists. Their median age was 33 years (IQR: 31, 38), and 8 (30%) were female. The median work experience was 6 years (IQR: 2, 11.5), and 14 (51%) had completed their training.

The third study phase aimed to investigate the potential preoperative usefulness of VR MRCP before CHE. Thirteen surgical trainees participated. Their median age was 31 years (IQR: 30, 34), and 7 (54%) were female (Table 1).

**Table 1:** Participant baseline characteristics, separated by study step.

	First phase	Second phase	Third phase
Total, *n*	34	27	13
Gender, *n* (%)			
Male	20 (58%)	19 (70%)	6 (46%)
Female	14 (42%)	8 (30%)	7 (54%)
Age, median [IQR]	37.5 [37, 45]	33 [31, 38]	31 [30, 34]
Years of experience, median [IQR]	11 [11, 17.25]	6 [2, 11.5]	5 [3.5, 6]
Finished training, *n* (%)	24 (71%)	14 (51%)	0 (0%)
Specialisation, *n* (%)			
Surgery	31 (91%)	22 (81%)	13 (100%)
Gastroenterology	0 (0%)	3 (11%)	0 (0%)
Radiology	3 (9%)	1 (4%)	0 (0%)
Internal medicine	0 (0%)	1 (4%)	0 (0%)

Abbreviations: IQR, Interquartile Range.

### First phase: VR tolerability and recognizability of biliary anatomy

All 34 (100%) participants were able to identify the gallbladder, cystic duct, common hepatic duct, common bile duct, and pancreatic duct. They all completed the VR testing without intervention or assistance from the staff. The VR application's performance was uninterrupted, with no technical failures.

The median SSQ score was 0.75 (IQR: 0, 3.5), indicating good tolerability, with only minimal discomfort. This was also supported by the absence of additional interventions or interruptions due to tolerability issues with the VR experience.

### Second phase: Comparison of conventional and VR MRCP and CBDS assessment

**Figure 4:**
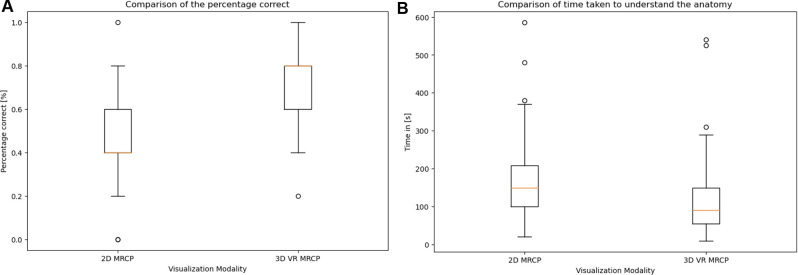
Box plots with 95% confidence intervals, with error bars showing (**A**) the number of correct answers as a percentage—left, conventional MRCP, and right, VR MRCP, and (**B**) the time needed to achieve sufficient understanding of the depicted anatomy—left, conventional MRCP, and right, VR MRCP.

The median time of image study was 90 s (IQR: 55, 150) in the VR MRCP group, and 150 s (IQR: 100, 208) in the conventional MRCP group, respectively (*p* < 0.001). The correct 3D printed MRCP model was selected by 72.7% of participants after VR MRCP viewing and by 49.6% of participants after conventional MRCP viewing, respectively (*p* < 0.001). The use of models was evenly distributed across both groups, measured with Pearson's chi-squared test (*p* = 0.6579) (Figure 4).

Age and years of experience showed no significant correlations with time to decision, time to correct decision, and correct answers. For conventional MRCP, the correlations of age and experience with time-to-decision, time-to-correct decision, and correct answers were *r* = −0.166 (*p* = 0.448), *r* = −0.314 (*p* = 0.143), and *r* = 0.254 (*p* = 0.219), respectively. For VR MRCP, the correlations of age and experience with time-to-decision, time-to-correct decision, and correct answers were *r* = −0.059 (*p* = 0.776), *r* = 0.176 (*p* = 0.397), and *r* = −0.247 (*p* = 0.244), respectively.

A total of 5 randomly selected scans of CBDS were shown to 17 participants in the VR system. The participants correctly identified the presence and localization of CBDS in 90.5% (77/85) of VR scans using the cutting plane.

### Third phase: Effect of VR MRCP on CHE

Thirteen participants performed CHEs after preoperatively studying the MR imaging of patients—8 with VR MRCP and 5 with conventional MRCP, respectively. In the VR MRCP and conventional MRCP groups, the median years of training were 5.5 (IQR: 2, 6.25) and 5 (IQR: 4, 5), respectively, and the number of previously performed CHEs was 26 (IQR: 8, 43) and 23 (IQR: 7, 33), respectively. The median times to study VR and conventional MRCP were 6.5 min (IQR: 5.75, 8.5) and 5 (IQR: 4, 7) min, respectively. The median operating times were 90.5 min (IQR: 72.5, 120) and 66 (IQR: 60, 70) min, CVS was achieved in all cases, and the median times to reach CVS were 43 min (IQR: 30, 46.5) and 34 (IQR: 21, 35) min, respectively. Intraoperative performance in the VR and conventional MRCP groups, measured with the GOALS score, resulted in self-assessment median scores of 17.5 (IQR: 14.75, 21.25) and 16.00 (IQR: 15.00, 20.00), respectively. Assessment by the supervising surgeon resulted in scores of 16 (IQR: 13, 22.25) and 11 (IQR: 10.75, 14.25), respectively. The 90-day morbidity and mortality rate was 0% (Table 2). These results should be viewed as exploratory.

**Table 2:** Results of direct comparison for preoperative preparation of LC with VR MRCP and conventional MRCP.

	Overall	Conventional MRPC	VR MRCP	*p*
*n*	13	5	8	
Age (years) (median [IQR])	31 [30, 34]	31 [30, 32]	32.50 [29.75, 34.50]	0.941
Year of training (median [IQR])	5 [2, 6]	5 [4, 5]	5.5 [2, 6.25]	0.599
Number of LC performed (median [IQR])	23 [8, 33]	23 [7, 33]	26 [8, 42.75]	0.66
Predicted difficulty (median [IQR])	4 [2, 7]	3 [1, 4]	4.5 [3.5, 7.25]	0.208
Previous surgery = yes (%)	4 (30.8)	2 (40)	2 (25)	1
Time for preparation (min) (median [IQR])	6 [5, 8]	5 [4, 7]	6.5 [5.75, 8.5]	0.267
Planned operative time (min) (median [IQR])	80 [70, 90]	90 [75, 90]	75 [67.5, 90]	0.225
Nassar grade (%)				0.152
1	2 (17)	2 (40)	0 (0)	
2	5 (41)	2 (40)	3 (43)	
3	5 (41)	1 (20)	4 (57)	
Operative time (min) (median [IQR])	78 [60, 120]	66 [60, 70]	90.5 [72.5, 120]	0.464
GOALS self assessment (median [IQR])	17 [15, 20]	16 [15, 20]	17.5 [14.75, 21.25]	0.659
GOALS examiner assessment (median [IQR])	16 [12, 22]	11 [11, 18]	16 [13, 22.25]	0.27
In-hospital complications = *n*	0	0	0	NA
Length of stay (days) (median [IQR])	2 [2, 2]	2 [2, 2]	2 [2, 2]	0.429

Median answers and interquartile ranges on the Likert scale, as well as percentage of positive responses (rating of 4 or 5 in positive and 1 or 2 in inversely formulated questions).

Abbreviations: CVS, critical view of safety; min, minutes.

### Analysis of the questionnaires

All participants completed pre- and post-test surveys: 34 in the first phase, and 27 in the second.

After the first phase, participants rated the overall VR experience as pleasant (100% positive responses) and useful for understanding anatomy (100%), and the VR system was seen as easy to handle (100%). Most patients deemed VR MRCP a potentially useful tool for decreasing treatment errors (94.1%), preparing for CHE (88%), and helping to decrease intraoperative complications, such as an injury to the common bile duct (91%).

The post-test survey after the second phase revealed that, after direct comparison of conventional to VR MRCP, understanding anatomy *via* VR MRCP was superior (92%). Most participants stated that VR facilitated choosing the correct printed 3D-model (89%). Subjects viewed VR as superior to conventional imaging for assessment of intrahepatic (67%) and extrahepatic (67%) biliary tract dilatation, CBDS (48%) and anatomic variants (96%), respectively (Table 3).

**Table 3:** Results of the questionnaire after the first study step (*n* = 34) and the second study step (*n* = 27), respectively.

Study Step	Question	Answers (Likert Scale) median [IQR]
First Study Step (*n* = 34)	Previously heard about VR	3 [3, 4] (100%)
	Easy handling	4 [4, 4] (91.2%)
	Handling is time consuming	2 [1, 2] (100%)
	Easy interaction	4 [3, 4] (100%)
	Easy to understand model / anatomy	4 [3.25, 4] (100%)
	Satisfied with time needed for VR	4 [3, 4] (100%)
	Would use VR again	4 [3, 4] (97.1%)
	Would recommend to other specialists	4 [3, 4] (97.1%)
	VR is helpful to understand the anatomy	4 [3, 4] (97.1%)
	VR usage can decrease errors in patients treatment	3 [3, 4] (94.1%)
	Enjoyable experience	4 [4, 4] (100%)
	Experience is a waste of time	1 [1, 1] (100%)
	VR can improve patient treatment	3 [3, 4] (91.2%)
	VR helps to anticipate problems during surgery	3 [3, 4] (88.2%)
	VR is less useful than expected	1 [1, 2] (97.1%)
	VR is useful for patient communication	3 [2.25, 4] (73.5%)
	VR is more useful than standard imaging	3 [2, 3] (67.6%)
Second Study Step (*n* = 27)	Usefulness of conventional MRCP	3 [3, 3] (85.2%)
	Usefulness of VR MRCP	3 [3, 4] (88.9%)

Median answers and interquartile ranges on the Likert scale, as well as percentage of positive responses (rating of 3 or 4 in positive and 1 or 2 in inversely formulated questions).

## Discussion

### Interpretation of the findings vs. other studies

Compared to previous studies using VR-enhanced cross-sectional imaging with manual sequencing (18, 19), the VR technology we used is able to import cross-sectional imaging datasets directly from the PACS archive. Imaging is displayed as an interactive 3D VR model in real time using freely adjustable transfer functions. This allows for unprecedented access to and routine use of VR-enhanced cross-sectional imaging.

This study included a wide range of participants in terms of age and clinical experience, ranging from novices to expert surgeons and medical professionals. This was important for ensuring the generalizability of the study results since surgeons working in hospitals (ranging from trainees to senior consultants) may be either digital natives or so-called “digital immigrants” (i.e., individuals who became acquainted with digital technologies in adulthood). Both groups performed comparably, which is encouraging regarding the usefulness and applicability of the technology. This study demonstrated that digital savviness or previous VR experience is not a prerequisite for successfully using VR MRCP, since there was no correlation between age, years of work experience, time needed, or correct answers.

Direct comparison between VR and conventional MRCP is difficult, and no templates exist for such a study. We therefore used 3D printed MRCP scans as testing grounds for understanding 3D biliary anatomy. The results showed a clear superiority of VR MRCP over conventional MRCP for the percentage of correct answers and the time needed to select the correct model. For study purposes, only scans displaying normal biliary anatomy were used. Assessing the value of VR MRCP in cases with aberrant anatomy might be an interesting question for future research.

The interpretation of the data for the third phase was challenging due to the relatively small number of participants, and no definite conclusions can be drawn at this point. Overall, the participants showed better intraoperative performance as measured by the GOALS score despite the cases being more difficult; however, these participants were slightly more experienced than their counterparts in the conventional MRCP group. To achieve balance and full understanding, a study with a larger number of participants will be needed.

Another approach to using VR technology as a teaching tool for surgical trainees is to make use of VR simulators, which are clearly distinguishable from the VR application used in this study (20). Unlike VR simulators, which resemble video games for teaching and training, we used VR technology to facilitate understanding of patient-specific anatomy and the transfer of this 3D knowledge to the operating theatre. These two approaches could complement each other and allow for an entirely new way of preparing surgeons for operations, and development of surgical simulation technology with these capabilities is ongoing. Augmented reality (AR) is another novel tool for clinical purposes, which we do not discuss in detail in this paper. A fusion of these approaches might be ideal for future applications (21, 22).

Another noteworthy result of the study was the relatively low number of correctly identified CBDS using VR MRCP (approximately 90%), although some study participants were relatively inexperienced and not trained radiologists. This result probably mirrors the degree of training of younger colleagues. In the literature, a 2002 study of 65 patients with MRCP showing CBDS revealed a similar detection rate (23).

### Limitations

This study has some limitations. The study intervention was known to the participants because blinding was not possible. Furthermore, the participants were recruited internally due to the availability of medical professionals with the background we planned to evaluate, which could have led to bias due to purely internal feedback. To counteract this weakness, the questionnaires were fully anonymized, and the study phases were performed separately for logistical reasons. However, between the study phases, the participants could have discussed their study experiences, potentially leading to peer-influenced bias in one or the other direction. In the third study phase, participants were aware that their performance was measured for study purposes, increasing the inherent risk of the Hawthorne effect (24) (i.e., altered behavior by study participants when they are aware of being observed). Another potential weakness of the study design is that we did not use any 3D reconstruction function in the conventional MRCP, possibly leading to weaker results in the conventional MRCP group. To counteract this effect, the two-dimensional (2D) VR MRCP software function was disabled in the third phase, clearly separating 2D and 3D experiences in the groups. Furthermore, not all institutions routinely use 3D reconstructions of MRCP imaging, and reconstructed MRCPs cannot be interacted with freely. Another relevant potential weakness concerns MRCP technology itself, which displays the water molecules and aqueous filling of the bile ducts and gallbladder, rather than the anatomical structures themselves; therefore, VR MRCP, like MRCP, provides an “indirect” image of biliary anatomy. For certain pathologies, such as CBDS, MRCP is inferior to invasive diagnostic procedures, such as intraoperative endoscopy or intraoperative cholangiography (25–27). Overall, MRCP is able to delineate anatomy reliably and present pathological findings with high accuracy. Furthermore, VR MRCP can only ever be as good as the underlying imaging and might not be usable with low-quality images due to movement artifacts and similar. Also, “hot-stuff” (novelty) bias, due to the pure novelty of VR MRCP, could have led the study participants to assess the technology more favorably. For large-scale clinical applications, most institutions do not routinely conduct MRCP imaging for elective CHEs, or only in selected cases. However, since cases selected using MRCP might be surgically more demanding, VR MRCP could be especially useful in such cases. Finally, the results of the third phase should be seen as exploratory due to the small number of participants and the short time frame and chance factor not adequately answering the question of whether VR MRCP leads to improved training of junior surgeons. It would be especially interesting to identify a subgroup of trainee surgeons who might benefit from VR MRCP. Less experienced colleagues might benefit most from this technology, since their 3D understanding of bile duct anatomy involves a steep learning curve.

### Implications for routine practice and further research

Since digitalization is rapidly advancing in healthcare, interactive digital tools for teaching and training have gained greater importance in the surgical field (3, 28). The results of this study provide the first evidence that teaching, training, ultimate intraoperative performance, and patient safety in CHEs could be improved in the future using VR MRCP. To further study these questions, a large, long-term prospective randomized controlled trial comparing both VR MRCP and conventional MRCP as preparation tools for trainee surgeons is needed. We have designed such a trial, and a pilot study to further establish perioperative parameters for assessing teaching efficacy is currently underway and is actively recruiting (Clinicaltrials.gov identifier NCT05169073). Should the pilot study show improved performance in surgeons who preoperatively study VR MRCP imaging prior to CHE, VR enhancement of cross-sectional imaging could become a key technology in surgical education, potentially rendering teaching efforts more effective and leading to shorter training periods for junior surgeons and ultimately improved intraoperative performance and patient safety. VR technology in combination with fluorescent cholangiography is an exciting novel approach to utilize VR intraoperatively to further improve safety of cholecystectomy (29). Attempts in this direction have been made, and the combination of preoperative VR exploration with intraoperative AR navigation merits further attention and development (30). In the long term, a combination of technologies, including VR operation simulations with real-life patient data, could revolutionize surgical training and catapult preoperative preparation into a completely new era, since operations could be practiced, and anatomy studied on simulators using real, interactive anatomical data.

## Conclusion

In conclusion, VR-enhanced MRCP proved to be understandable and tolerable, and it allowed for a faster and more accurate understanding of 3D biliary anatomy than conventional MRCP. Furthermore, preoperative study of VR MRCP potentially leads to improved surgical performance of trainee surgeons in CHE.

## Data Availability

The raw data supporting the conclusions of this article will be made available by the authors, without undue reservation.

## References

[1] Beheiry ME Doutreligne S Caporal C Ostertag C Dahan M Masson JB. Virtual reality: beyond visualization. J Mol Biol. (2019) 431(7):1315–21. 10.1016/j.jmb.2019.01.033 30738026

[2] Maloca PM de Carvalho JER Heeren T Hasler PW Mushtaq F Mon-Williams M High-performance virtual reality volume rendering of original optical coherence tomography point-cloud data enhanced with real-time ray casting. Transl Vis Sci Technol. (2018) 7(4):2. 10.1167/tvst.7.4.2 PMC6038772 30002949

[3] Bernardo A. Virtual reality and simulation in neurosurgical training. World Neurosurg. (2017) 106:1015–29. 10.1016/j.wneu.2017.06.140 28985656

[4] Fahrner R Turina M Neuhaus V Schöb O. Laparoscopic cholecystectomy as a teaching operation: comparison of outcome between residents and attending surgeons in 1,747 patients. Langenbecks Arch Surg. (2012) 397(1):103–10. 10.1007/s00423-011-0863-y 22012582

[5] Adams DB. The importance of extrahepatic biliary anatomy in preventing complications at laparoscopic cholecystectomy. Surg Clin North Am. (1993) 73(4):861–71. 10.1016/S0039-6109(16)46089-5 8378825

[6] Flum DR. Bile duct injury during cholecystectomy and survival in medicare beneficiaries. JAMA. (2003) 290(16):2168. 10.1001/jama.290.16.2168 14570952

[7] Strasberg SM Brunt LM. Rationale and use of the critical view of safety in laparoscopic cholecystectomy. J Am Coll Surg. (2010) 211(1):132–8. 10.1016/j.jamcollsurg.2010.02.053 20610259

[8] Reitano E de’Angelis N Schembari E Carrà MC Francone E Gentilli S Learning curve for laparoscopic cholecystectomy has not been defined: a systematic review. ANZ J Surg. (2021) 91(9):e554–60. Verfügbar unter. 10.1111/ans.17021 34180567 PMC8518700

[9] Voitk AJ Tsao SGS Ignatius S. The tail of the learning curve for laparoscopic cholecystectomy. Am J Surg. (2001) 182(3):250–3. 10.1016/S0002-9610(01)00699-7 11587686

[10] Bennett CL. The learning curve for laparoscopic cholecystectomy. Am J Surg. (1995) 170(1):55–9. 10.1016/S0002-9610(99)80252-9 7793496

[11] Faulkner L. Beyond the five-user assumption: Benefits of increased sample sizes in usability testing. Behav Res Methods Instrum Comput. (2003) 35(3):379–83. 10.3758/bf03195514 14587545

[12] Kennedy RS Fowlkes JE Berbaum KS Lilienthal MG. Use of a motion sickness history questionnaire for prediction of simulator sickness. Aviat Space Env Med. (1992) 63(7):588–93. PMID: 1616434

[13] Gupta N Ranjan G Arora MP Goswami B Chaudhary P Kapur A Validation of a scoring system to predict difficult laparoscopic cholecystectomy. Int J Surg. (2013) 11(9):1002–6. 10.1016/j.ijsu.2013.05.037 23751733

[14] Nassar AHM Ng HJ Wysocki AP Khan KS Gil IC. Achieving the critical view of safety in the difficult laparoscopic cholecystectomy: a prospective study of predictors of failure. Surg Endosc. (2021) 35(11):6039–47. 10.1007/s00464-020-08093-3 33067645 PMC8523408

[15] Sanford DE Strasberg SM. A simple effective method for generation of a permanent record of the critical view of safety during laparoscopic cholecystectomy by intraoperative “doublet” photography. J Am Coll Surg. (2014) 218(2):170–8. 10.1016/j.jamcollsurg.2013.11.003 24440064

[16] Vassiliou MC Feldman LS Andrew CG Bergman S Leffondré K Stanbridge D A global assessment tool for evaluation of intraoperative laparoscopic skills. Am J Surg. (2005) 190(1):107–13. 10.1016/j.amjsurg.2005.04.004 15972181

[17] Nebiker CA Baierlein SA Beck S von Flüe M Ackermann C Peterli R. Is routine MR cholangiopancreatography (MRCP) justified prior to cholecystectomy? Langenbecks Arch Surg. (2009) 394(6):1005–10. 10.1007/s00423-008-0447-7 19084990

[18] Kenngott HG Pfeiffer M Preukschas AA Bettscheider L Wise PA Wagner M IMHOTEP: cross-professional evaluation of a three-dimensional virtual reality system for interactive surgical operation planning, tumor board discussion and immersive training for complex liver surgery in a head-mounted display. Surg Endosc. (2022) 36(1):126–34. 10.1007/s00464-020-08246-4 PMC8741674 33475848

[19] Lin Q Xu Z Li B Baucom R Poulose B Landman BA Immersive virtual reality for visualization of abdominal CT. Medical imaging 2013: Image perception, observer performance, and technology assessment. (2013). 10.1117/12.2008050 PMC3877248 24386547

[20] Yiannakopoulou E Nikiteas N Perrea D Tsigris C. Virtual reality simulators and training in laparoscopic surgery. Int J Surg. (2015) 13:60–4. 10.1016/j.ijsu.2014.11.014 25463761

[21] Faludi B Zoller EI Gerig N Zam A Rauter G Cattin P. Direct visual and haptic volume rendering of medical data sets for an immersive exploration in virtual reality. In: Medical image computing and computer assisted intervention. Cham: Springer; (MICCAI 2019). Lecture Notes in Computer Science; Bd. 11768). Verfügbar unter: 10.1007/978-3-030-32254-0_4

[22] Zoller EI Faludi B Gerig N Jost GF Cattin PC Rauter G. Force quantification and simulation of pedicle screw tract palpation using direct visuo-haptic volume rendering. Int J Comput Assist Radiol Surg. (2020) 15(11):1797–805. 10.1007/s11548-020-02258-0 32959159 PMC7603448

[23] Simone M Mutter D Rubino F Dutson E Roy C Soler L Three-dimensional virtual cholangioscopy: a reliable tool for the diagnosis of common bile duct stones. Ann Surg. (2004) 240(1):82–8. 10.1097/01.sla.0000129493.22157.b7 15213622 PMC1356378

[24] McCambridge J Witton J Elbourne DR. Systematic review of the Hawthorne effect: new concepts are needed to study research participation effects. J Clin Epidemiol. (2014) 67(3):267–77. 10.1016/j.jclinepi.2013.08.015 24275499 PMC3969247

[25] Richard F Boustany M Britt LD. Accuracy of magnetic resonance cholangiopancreatography for diagnosing stones in the common bile duct in patients with abnormal intraoperative cholangiograms. Am J Surg. (2013) 205(4):371–3. 10.1016/j.amjsurg.2012.07.033 23518180

[26] Polistina FA. Accuracy of magnetic resonance cholangiography compared to operative endoscopy in detecting biliary stones, a single center experience and review of literature. World J Radiol. (2015) 7(4):70. 10.4329/wjr.v7.i4.70 25918584 PMC4404370

[27] Shen Z Munker S Zhou B Li L Yu C Li Y. The accuracies of diagnosing pancreas divisum by magnetic resonance cholangiopancreatography and endoscopic ultrasound: a systematic review and meta-analysis. Sci Rep. (2016) 6(1):35389. 10.1038/srep35389 27734952 PMC5062127

[28] Humm G Harries RL Stoyanov D Lovat LB. Supporting laparoscopic general surgery training with digital technology: the United Kingdom and Ireland paradigm. BMC Surg. (2021) 21(1):123. 10.1186/s12893-021-01123-4 33685437 PMC7941971

[29] Ishizawa T Bandai Y Ijichi M Kaneko J Hasegawa K Kokudo N. Fluorescent cholangiography illuminating the biliary tree during laparoscopic cholecystectomy. Br J Surg. (2010) 97(9):1369–77. 10.1002/bjs.7125 20623766

[30] Diana M Soler L Agnus V D’Urso A Vix M Dallemagne B Prospective evaluation of precision multimodal gallbladder surgery navigation: virtual reality, near-infrared fluorescence, and X-ray-based intraoperative cholangiography. Ann Surg. (2017) 266(5):890–7. 10.1097/SLA.0000000000002400 28742709

